# Temperature Compensation Method for Tunnel Magnetoresistance Micro-Magnetic Sensors Through Reference Magnetic Field

**DOI:** 10.3390/mi15101271

**Published:** 2024-10-20

**Authors:** Tao Kuai, Qingfa Du, Jiafei Hu, Shilong Shi, Peisen Li, Dixiang Chen, Mengchun Pan

**Affiliations:** 1 College of Intelligence Science and Technology, National University of Defense Technology, Changsha 410073, China; ktao9804@163.com (T.K.); garfield_nudt@163.com (J.H.); shishilong22@nudt.edu.cn (S.S.); lips13@163.com (P.L.); chendixiang@163.com (D.C.); pmc_nudt@vip.163.com (M.P.); 2 Northwest Institute of Mechanical and Electrical Engineering, Xianyang 712099, China

**Keywords:** TMR micro-magnetic sensor, temperature compensation, reference magnetic source, sensitivity calibration

## Abstract

The sensitivity of Tunnel Magnetoresistance (TMR) sensors is characterized by significant temperature drift and poor sensitivity drift repeatability, which severely impairs measurement accuracy. Conventional temperature compensation techniques are often hindered by low compensation precision, inadequate real-time performance, and an inability to effectively address the issue of poor repeatability in temperature drift characteristics. To overcome these challenges, this paper introduces a novel method for suppressing temperature drift in TMR sensors. In this method, an alternating reference magnetic field is applied to TMR sensors, and the output amplitude at the frequency of the reference magnetic field is calculated to compensate the sensitivity temperature drift in real time. Temperature characteristic tests were conducted in a non-magnetic temperature test chamber, and the results revealed that the proposed method significantly reduced the TMR sensitivity drift coefficient from 985.39 ppm/°C to 59.08 ppm/°C. Additionally, the repeatability of sensitivity temperature characteristic curves was enhanced, with a reduction in root mean square error from 0.84 to 0.21. This approach effectively mitigates temperature-induced sensitivity drift without necessitating the use of a temperature sensor, and has the advantages of real-time performance and repeatability, providing a new approach for the high-precision temperature drift suppression of TMR.

## 1. Introduction

Tunnel Magnetoresistance (TMR) sensors represent a novel category of magnetic sensing devices distinguished by their high sensitivity, low power consumption, and compact size. These attributes render them particularly advantageous for applications across various domains, including automotive, healthcare, industrial automation, and both military and civilian sectors [1,2,3,4]. However, the significant temperature drift observed in TMR sensors poses a considerable challenge, severely constraining their applicability in high-precision measurement contexts. Variations in the ambient temperature of a TMR sensor lead to fluctuations in TMR [5,6,7]. Specifically, as the temperature rises, the coercivity difference between the two ferromagnetic layers within the magnetic tunnel junction diminishes, resulting in the alignment of the magnetic moments [8]. Concurrently, oxidation occurs on the surface of the ferromagnetic layer, which induces spin scattering. As temperature increases, the spin-flip scattering associated with the oxide layer becomes more pronounced, thereby reducing the spin diffusion length of the charge carriers. This reduction subsequently diminishes the spin polarization probability of the ferromagnetic layers, thereby weakening the magnetoresistive effect and decreasing the magnitude of TMR, which exhibits a negative temperature characteristic [9,10]. TMR sensors employ a Wheatstone bridge configuration [11,12]. However, variations in manufacturing processes and material properties result in inconsistencies in the temperature drift characteristics of the four magnetoresistances that constitute the bridge. Consequently, these sensors exhibit significant temperature drift in environments with fluctuating temperatures [13]. The temperature drift associated with TMR sensors can be categorized into sensitivity drift and zero-point drift [14,15]. Unlike other sensor types, the temperature drift characteristics of TMR sensors are affected by environmental factors, including magnetic fields, which introduce multiple sources of drift, diminish repeatability, and contribute to nonlinearity. These attributes constrain the efficacy of conventional compensation methods [16]. Therefore, implementing temperature compensation is essential for TMR sensors to improve the accuracy of measurements within sensor systems.

Currently, temperature compensation methods for TMR magnetic sensors are primarily categorized into hardware and software compensation approaches. Hardware compensation typically involves the selection of components with temperature characteristics that are opposite to those of the TMR sensor, as well as the design of appropriate circuit structures to achieve effective compensation. Lei et al. [17] proposed a TMR temperature compensation circuit that incorporates a thermistor with a negative temperature coefficient. As the temperature increases, the resistance of the thermistor decreases, thereby maintaining the output voltage at a constant value. This method reduced the TMR sensitivity temperature coefficient from 1780 ppm/°C to 504 ppm/°C. Although this approach provides a certain degree of suppression of temperature drift, its effectiveness in compensation is limited, rendering it insufficient for high-precision measurement requirements. Zhou [18] designed a temperature-sensitive voltage source using a thermistor to supply power to a TMR sensor. As the temperature rises, the resistance of the thermistor decreases, causing an increase in the supply voltage to the TMR chip, which offsets the output error caused by temperature drift. After compensation, the maximum bias voltage was reduced from −0.053 V to −0.015 V. However, due to the inherent limitations of the thermistor, the compensation circuit is unable to operate in environments where the temperature falls below 25 °C. Hardware compensation can also be achieved through the implementation of a bridge circuit, wherein the reference resistor may be constituted by the TMR sensor itself [19]. Le et al. [20] introduced an innovative compensation methodology utilizing two TMR magnetic sensors sourced from the same manufacturer, model, and production batch. These sensors were configured to establish a signal measurement channel alongside a reference magnetic field measurement channel. Both sensors utilized an identical circuit for signal processing, thereby obviating the need for an additional temperature sensor and reducing the sensitivity error to within ±0.6%. However, ensuring that the characteristics of the two TMR sensors are perfectly identical remains a significant challenge, which may lead to measurement inaccuracies. Liu et al. [21] proposed a similar approach, using two identical TMR chips as a measurement channel and a standard channel, respectively. The output voltage of TMR in the standard channel with reference magnetic field was used as calibration voltage to correct the sensitivity temperature drift of the TMR measuring chip. Finally, the sensitivity temperature drift of the sensor was reduced from −369 ppm/°C to −81 ppm/°C. Although hardware compensation circuits are simple and relatively easy to implement, their effectiveness is constrained by the precision of the chosen components. Consequently, the accuracy of compensation remains relatively low, and this approach fails to resolve the issues of nonlinearity and poor repeatability associated with TMR temperature drift [22]. Software compensation involves an initial assessment of the temperature characteristics of the TMR sensor, followed by the application of a series of algorithms to construct a temperature characteristic curve. This curve is subsequently integrated with a temperature sensor and a digital microprocessor to facilitate compensation [23]. Li [24] explored the temperature characteristics of a TMR sensor utilizing an interface circuit in conjunction with a temperature sensor, which transmitted the data to a digital microprocessor. By employing the least squares method, a fitted temperature characteristic curve was derived, resulting in a reduction in the temperature coefficient from −1100 ppm/°C to −117 ppm/°C. However, the fixed temperature characteristic curve obtained through least squares fitting frequently fails to provide effective compensation for temperature drift, exhibiting poor repeatability. Xie et al. [25] employed an enhanced B-spline interpolation algorithm for the temperature compensation of magnetoresistance sensors, which resulted in a reduction in the number of sampling points and an increase in computational speed. Nevertheless, this calibration method cannot reflect real-time changes in the sensor’s sensitivity, limiting the effectiveness of the compensation. While software-based compensation offers greater flexibility and higher precision compared to hardware compensation, especially in addressing nonlinear temperature drift, it has the following limitations: first, it requires support from temperature sensors and processors; second, the accuracy and response speed of the temperature sensor, as well as its positioning, can affect the overall compensation precision, leading to poor real-time performance; and, finally, it fails to address the issue of inconsistent temperature drift characteristics. The fixed temperature characteristic curve obtained through curve fitting may provide good compensation for a particular output, but it is difficult to ensure effective compensation across the multiple outputs of the TMR sensor, thus limiting the overall compensation capability.

In summary, traditional compensation methods are characterized by low precision and inadequate real-time performance, and there is currently no effective solution to the issue of poor repeatability associated with temperature drift characteristics. To address these challenges, this paper proposes a temperature drift suppression method that utilizes an alternating reference magnetic source, specifically targeting the sensitivity temperature drift of TMR sensors. A real-time temperature compensation model is developed based on the alternating reference magnetic source. By analyzing the variations in the output amplitude of the alternating signal, the sensitivity changes induced by temperature fluctuations are quantified. This approach effectively mitigates the challenges posed by the multiple sources of temperature drift, poor repeatability, and nonlinearity that compromise the efficacy of traditional temperature compensation methods. Furthermore, it eliminates the necessity for establishing a high-precision TMR temperature drift model, thereby simplifying the compensation process and enhancing its applicability in practical scenarios.

## 2. Principle and Method

**Figure 1:**
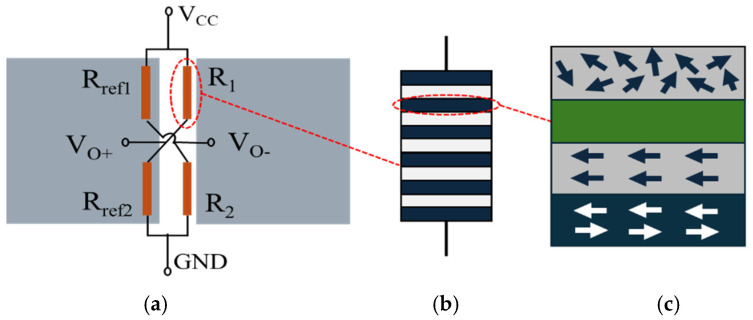
Structural diagram of TMR micro-magnetic sensor: (**a**) TMR micro-magnetic sensor; (**b**) magnetic resistor; (**c**) magnetic tunnel junction.

The TMR magnetic sensor is structured as a Wheatstone bridge, which incorporates magnetic concentrators and four magnetoresistance elements (Figure 1). The magnetoresistance elements consist of multiple magnetic tunnel junctions arranged in series, facilitating the flow of spin-polarized electrons. The air gap between the two magnetic flux concentrators functions as the area for magnetic field concentration. Magnetoresistance elements $R_{ref1}$ and $R_{ref2}$ are positioned within the shielded region at the base of the magnetic concentrators, serving as reference resistors with fixed resistance values. In contrast, the remaining two magnetoresistance elements ($R_{1}$ and $R_{2}$) are located in the air gap between the magnetic concentrators, where their resistance values fluctuate in response to variations in the magnetic field within the gap. The output of the TMR magnetic sensor is determined by changes in the external magnetic field, as expressed by the following equation:

(1) $$V_{out}=\frac{1}{2\frac{R_{0}}{\Delta R}+1}V_{CC},$$

where $V_{out}$ is the output voltage of the TMR magnetic sensor, $V_{cc}$ is the power supply voltage of the TMR sensor, the resistance value of the reference reluctance is $R_{0}$, the initial resistance value of the two sensitive reluctance is $R_{0}$, and the variation is Δ*R*.

**Figure 2:**
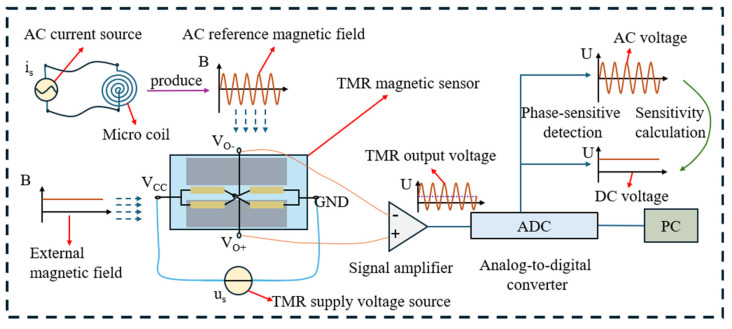
Temperature compensation principle of TMR magnetic sensor based on AC reference magnetic source.

The sensitivity of a TMR magnetic sensor is characterized as the ratio of the change in output to the corresponding change in input. Variations in temperature can lead to sensitivity drift in the sensor. The sensitivity drift of TMR magnetic sensors ranges from several hundred to several thousand ppm/°C, and traditional temperature compensation methods provide limited effectiveness, making them insufficient to meet the demands of high-precision measurements. In this method, an alternating current (AC) source is employed to apply a consistent AC to a miniature coil, which in turn generates a stable AC magnetic field at the same frequency. This magnetic field, in conjunction with the external magnetic field, exerts an effect on the TMR sensor. The output signal from the TMR sensor is subsequently amplified using an amplifier, and the resulting signal is collected by a data acquisition card. Through the process of phase-sensitive detection, an AC voltage signal corresponding to the reference magnetic field frequency is isolated. By analyzing this AC output signal, the real-time sensitivity drift of the TMR sensor can be determined. This drift is then utilized to adjust the output of the TMR sensor in response to the target magnetic field, thereby facilitating temperature compensation for the TMR sensor. The principle underlying the temperature drift compensation of the TMR sensor is depicted in Figure 2.

The output voltage of a TMR magnetic sensor operating in the linear region can be expressed as:

(2) $$U_{O}=SB+V_{0},$$

where S is the sensitivity of the TMR magnetic sensor, B is the magnetic field around the sensor, and $V_{0}$ is the sensor’s offset voltage. The effect of temperature on the TMR magnetic sensor primarily manifests in shifts in both the sensitivity and the offset voltage. When the temperature around the TMR sensor changes, the output voltage is given by:

(3) $$U_{O}=S(T)B+V_{0}(T),$$

When an alternating current magnetic field $B_{ac}$ with a frequency $f_{0}$ and amplitude $B_{ac0}$ within the sensor’s bandwidth is applied, the magnetic field at the TMR magnetic sensor can be expressed as:

(4) $$B=B_{ac}+B_{f}=B_{ac0}\cos(\frac{t}{f_{0}})+B_{f},$$

where $B_{f}$ represents the external magnetic field to be measured. At this point, the output voltage of the TMR magnetic sensor is given by:

(5) $$U_{O}=S(T)[B_{ac0}\cos(\frac{t}{f_{0}})+B_{f}]+V_{0}(T),$$

The AC output voltage at frequency $f_{0}$ is:

(6) $$U_{OAC}=A\cos(\frac{t}{f_{0}})=S(T)B_{ac0}\cos(\frac{t}{f_{0}}),$$

where A is the amplitude of the AC signal at frequency $f_{0}$. By using synchronous detection techniques, the AC amplitude at $f_{0}$ can be obtained, and the AC sensitivity of the TMR magnetic sensor is given by:

(7) $$S_{AC}(T)=\frac{A}{B_{ac0}},$$

The DC output voltage $U_{ODC}$ is:

(8) $$U_{ODC}=S_{DC}(T)B_{f}+V_{0}(T),$$

The DC sensitivity of the TMR magnetic sensor is given by:

(9) $$S_{DC}(T)=\frac{U_{ODC}-V_{0}(T)}{B_{f}},$$

The bias voltage also varies with temperature, so the zero bias voltage value needs to be calibrated in advance when calculating sensitivity. With the sensitivity $S_{0}$ at 0 °C as the reference sensitivity, the sensitivity compensation formula is:

(10) $$S(T)=S_{0}\times \frac{S_{AC}(T)}{S_{DC}(T)},$$

**Figure 3:**
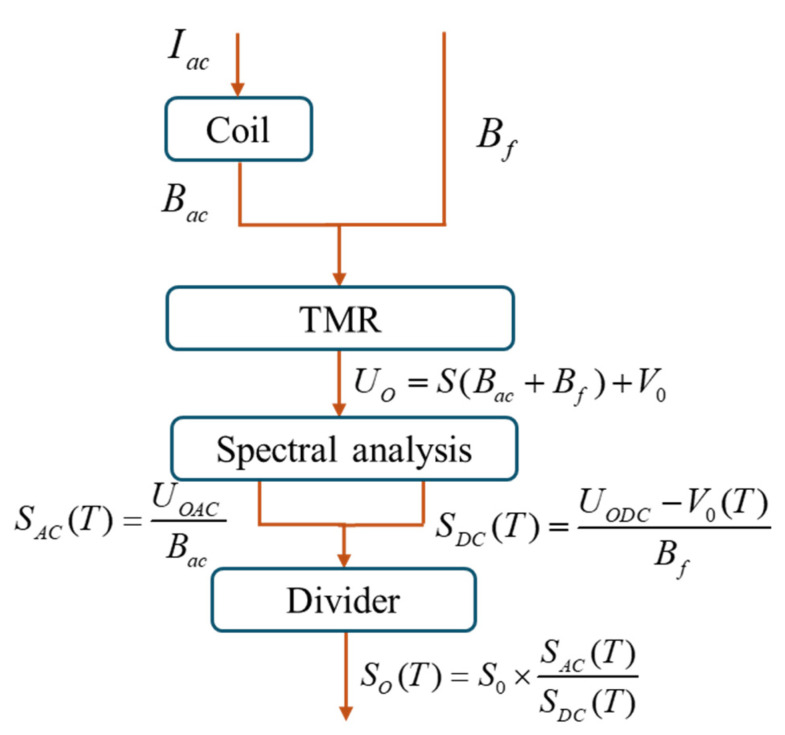
Flow chart of AC reference magnetic source compensation method.

The temperature compensation schematic for the TMR magnetic sensor based on an AC reference magnetic source is shown in Figure 3.

## 3. Test System

**Figure 4:**
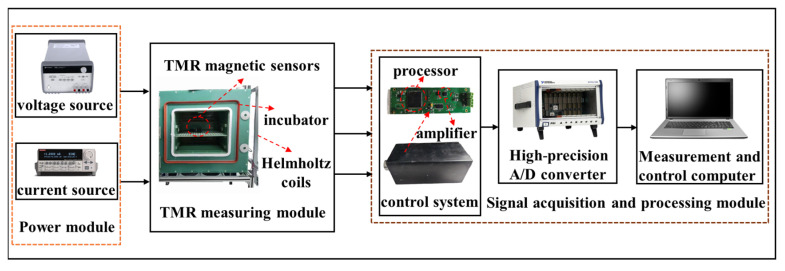
TMR magnetic sensor sensitivity temperature drift test system.

The overall schematic of the TMR temperature compensation system used in this experiment is shown in Figure 4.

The power supply system comprises both a direct current (DC) voltage source and an AC source. The DC voltage source utilized is KEITHLEY 3649A, which is capable of delivering a DC voltage within the range of 0 V to 35 V to power the TMR chip. The AC source, KEITHLEY 6221, is capable of outputting an AC ranging from 0 mA to 105 mA to energize the coil, thereby generating the alternating reference magnetic field. The TMR sensor system comprises a TMR chip, a temperature sensor, and a micro coil. In this design, the TMR magnetic sensor employs a Wheatstone full-bridge configuration, which consists of four high-sensitivity TMR elements exhibiting a sensitivity of approximately 100 mV/V/Oe. To monitor the temperature variations of the TMR sensor, a temperature sensor is integrated into the TMR circuit for this experiment, although the temperature compensation method based on the alternating reference magnetic source does not necessitate the inclusion of a temperature sensor. The temperature sensor is TMP116, which has the characteristics of high precision and low power consumption, and can work normally in the range of −55 °C to 125 °C. The micro coil is integrated onto the circuit board, and an alternating current is supplied to it. According to Faraday’s law of electromagnetic induction, the coil generates an alternating magnetic field at the same frequency. The excitation factor of the micro coil is 3750 nT/mA. By applying an AC with an amplitude of 8 mA and a frequency of 70 Hz through the KEITHLEY 6221 AC signal generator, an alternating magnetic field with an amplitude of 30,000 nT can be produced at this frequency. The signal acquisition and processing system comprises a low-noise amplifier circuit utilizing the AD8429, a processor, a 24-bit high-precision Analog-to-Digital Converter (ADC), and a LabVIEW 2018 testing system. The processor is tasked with transmitting real-time temperature data obtained from the temperature sensor to the host computer. The acquired voltage output encompasses both the reference AC voltage signal and the external DC signal. Through the application of LabVIEW integrated testing software, the magnitude of AC signal is computed via phase-sensitive detection, and the sensitivity value of TMR sensor is ascertained.

**Figure 5:**
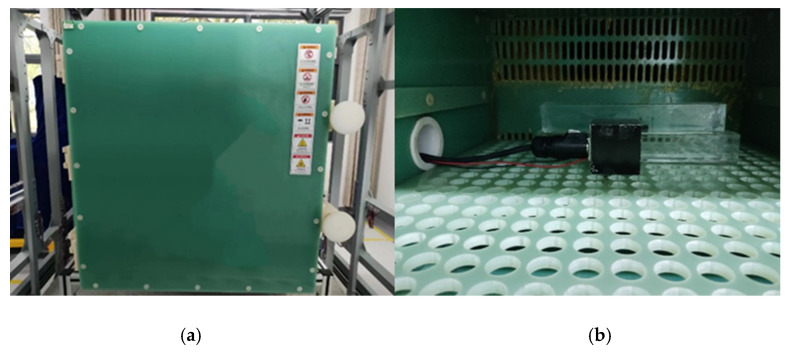
Temperature test diagram of TMR micro-magnetic sensor: (**a**) non-magnetic temperature box; (**b**) TMR magnetic sensor system.

The testing environment for this study is shown in Figure 5.

**Figure 6:**
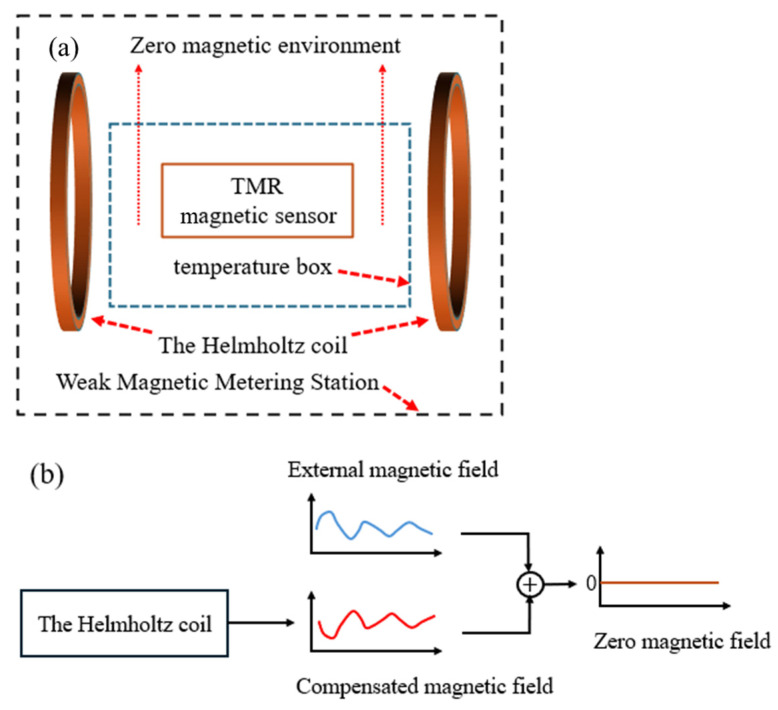
Schematic diagram of the process for achieving a zero magnetic field environment around the TMR sensor. (**a**) Schematic diagram of the devices that achieve a zero magnetic field. (**b**) Schematic diagram of the process to achieve a zero magnetic field.

The experiment was carried out in a magnetically neutral environment. The temperature experiment was carried out at the First-Class Weak Magnetic Metering Station. The temperature chamber utilized in the experiment was constructed from non-magnetic materials. Circulating hot air was used to heat the TMR magnetic sensor to prevent the generation of any interfering magnetic fields during temperature fluctuations. The temperature chamber was situated within a Helmholtz coil, with the TMR magnetic sensor strategically positioned at the center of the chamber. The sensitive axis of the sensor was aligned with the direction of the excitation field produced by the Helmholtz coil, thereby ensuring that the angle between the magnetic field generated by the Helmholtz coil and the sensor’s sensitive axis was maintained at zero degrees. The Helmholtz coil produced an equal and opposite magnetic field to mitigate any interference fields surrounding the chamber, including the geomagnetic field as well as the equipment magnetic field, thereby ensuring that the chamber functioned within a zero magnetic environment. The specific principle of making the magnetic field around the TMR sensor zero is shown in Figure 6. A fluxgate magnetometer was employed to measure the magnetic field within the temperature chamber. The output magnetic field value of the Helmholtz coil was adjusted according to the output value of the fluxgate. When the fluxgate output value is 0 nT, the TMR sensor is in a non-magnetic environment. Finally, the true magnetic field inside the temperature chamber in the weak magnetic metering station was less than 1 nT, which did not affect the accuracy of the experimental results. The temperature range for the experiment was established between 0 °C and 125 °C.

## 4. Experiment

To verify that the temperature compensation method based on an AC reference magnetic source can address issues related to low compensation accuracy due to nonlinear sensitivity drift and poor repeatability, this study conducted an experiment on the temperature characteristics of TMR sensitivity. The experiment measured TMR sensitivity at different temperatures, plotted sensitivity characteristic curves, and analyzed the repeatability and linearity of the sensitivity curves across different temperatures. To validate the authenticity of the proposed compensation method, experiments were conducted to test the AC and DC sensitivities of the TMR magnetic sensor. Sensitivity characteristic curves for both AC and DC measurements at the same time were plotted, and an analysis of the relationship between the two sensitivities was performed. The temperature compensation method based on the AC reference magnetic source was used to correct the nonlinear and poorly repeatable sensitivity drift characteristics. The results were compared with those obtained using traditional methods, demonstrating the effectiveness of the proposed approach.

### 4.1. Nonlinearity and Repeatability Experiments

**Figure 7:**
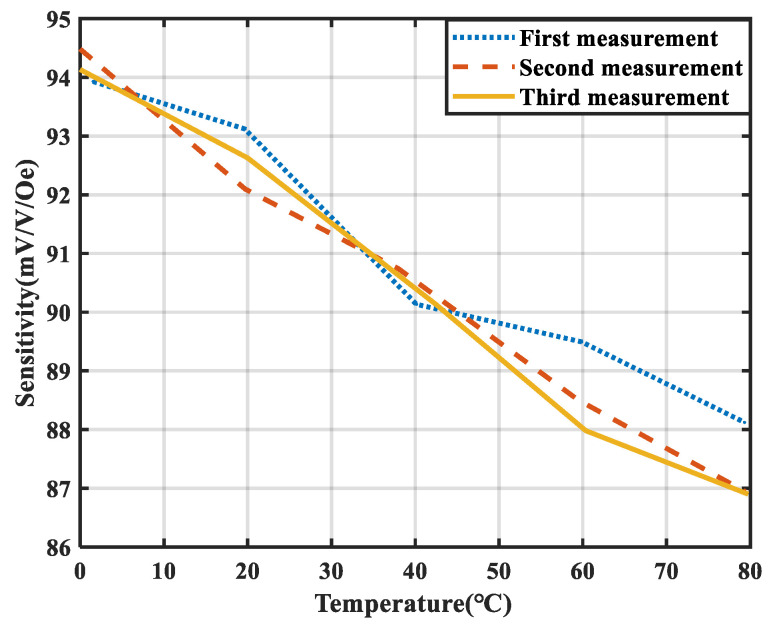
Temperature characteristic curve of the sensitivity of the TMR sensor.

The sensitivity drift of the TMR magnetic sensor under varying temperature conditions significantly impacts the accuracy of its measurements. The temperature of the environmental chamber was adjusted within the range of 0 °C to 80 °C, with measurements performed at 20 °C intervals. Three sets of independent and repeatable experiments were conducted under identical conditions to assess the sensitivity. The test results are shown in Figure 7.

It can be observed that as the temperature increases, the sensitivity of the TMR magnetic sensor fluctuates in the range 86.63 mV/V/Oe~94.481 mV/V/Oe. Over the temperature range of 0 °C to 80 °C, the deviation in TMR sensitivity reaches 8.67%. The linearity of the three sensitivity–temperature characteristic curves is relatively poor, indicating that traditional hardware compensation methods cannot achieve effective compensation. Furthermore, the poor repeatability of the TMR sensor’s sensitivity–temperature drift characteristic curves renders the use of fixed fitting equations for temperature compensation in conventional software methods impractical.

### 4.2. AC and DC Magnetic Field Sensitivity Consistency Experiment

**Figure 8:**
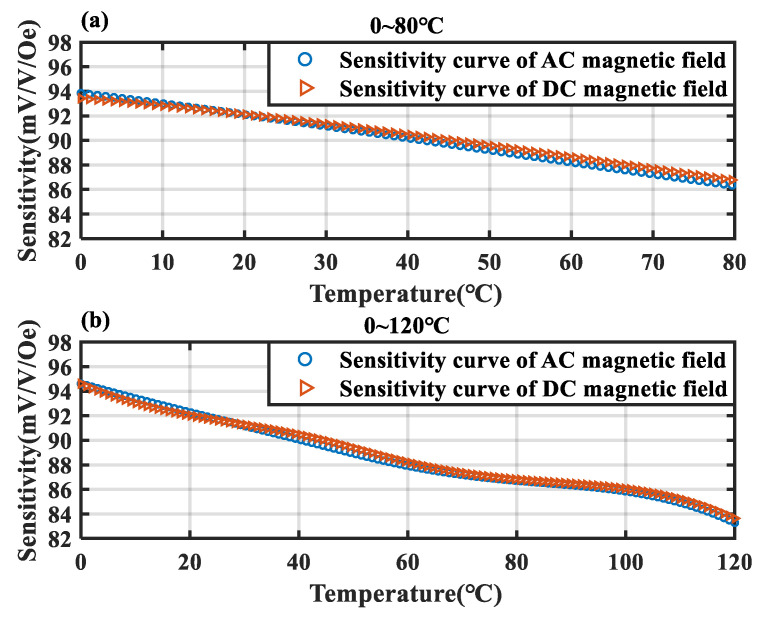
Testing curves of AC sensitivity and DC sensitivity. (**a**) AC and DC sensitivity–temperature characteristic curves in the range of 0–80 °C. (**b**) AC and DC sensitivity–temperature characteristic curves in the range of 0–120 °C.

To verify the validity of the proposed method, an experiment was conducted to assess the consistency of AC and DC magnetic field sensitivities. The temperature chamber was initially set to 0 °C and maintained. Once thermal equilibrium between the TMR sensor and the chamber was achieved, an alternating current of 8 mA was applied to the coil using an AC source. After the temperature sensor readings stabilized, an additional 0.1 mA of AC was introduced for 1 s and then removed. Subsequently, a 0.1 mA DC was applied for 1 s and then removed. The variations in the TMR sensor’s AC and DC outputs were used to calculate the AC and DC sensitivities. The temperature of the chamber was adjusted, and TMR sensitivity at different temperatures was recorded. The results are presented in Figure 8.

Figure 8a shows the AC and DC sensitivity curves of the TMR magnetic sensor across the temperature range from 0 °C to 80 °C. Figure 8b shows the sensitivity curves across the temperature range of 0 °C to 120 °C. It can be observed that the DC and AC sensitivities of the TMR magnetic sensor remain consistent across temperature variations, with the maximum deviation not exceeding 0.44 mV/V/Oe. Therefore, the method of replacing DC sensitivity with AC sensitivity calculated by an AC reference magnetic source in this scheme is feasible.

### 4.3. Temperature Compensation Experiment Based on the AC Reference Magnetic Source

A 10,000 nT DC external magnetic field was applied to the TMR sensor using Helmholtz coils. The temperature chamber was initially set to 0 °C, and after the temperature stabilized, the TMR output voltage was recorded. Sensitivity compensation was performed using the TMR sensitivity measured at 0 °C as the reference. The chamber temperature was then varied between 0 °C and 120 °C, and the output voltage of the TMR magnetic sensor was recorded. The final sensitivity test results are presented in Table 1.

**Figure 9:**
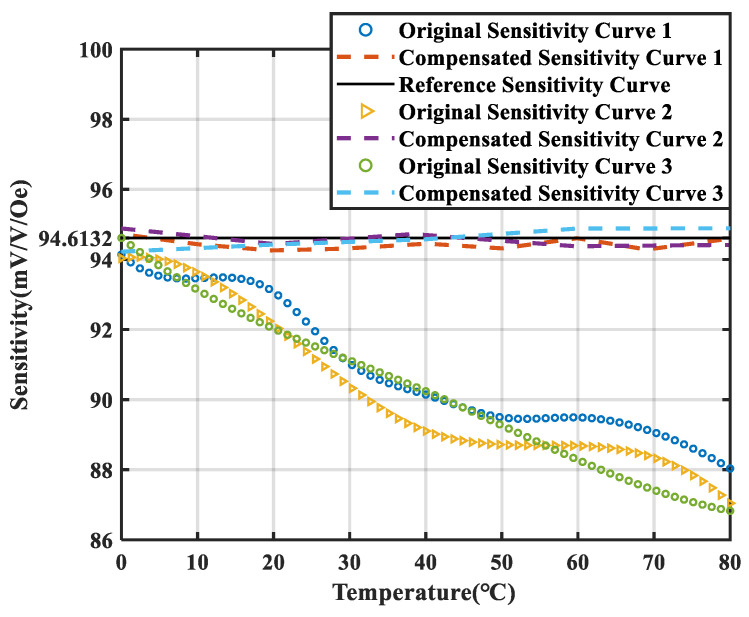
The different sensitivity drift curves in the range of 0–80 °C and the curves obtained by temperature compensation of the AC reference magnetic source.

According to the data in this table, the reference sensitivity value at 0 °C was 94.61 mV/V/Oe. After compensation with the AC reference magnetic source, the TMR sensitivity drift coefficient decreased from 985.39 ppm/°C to 59.08 ppm/°C. Three repeated experiments were conducted continuously over the temperature range of 0 °C to 80 °C. The temperature compensation method based on the reference magnetic source was applied to the TMR temperature characteristic curves, and the results are shown in Figure 9.

**Figure 10:**
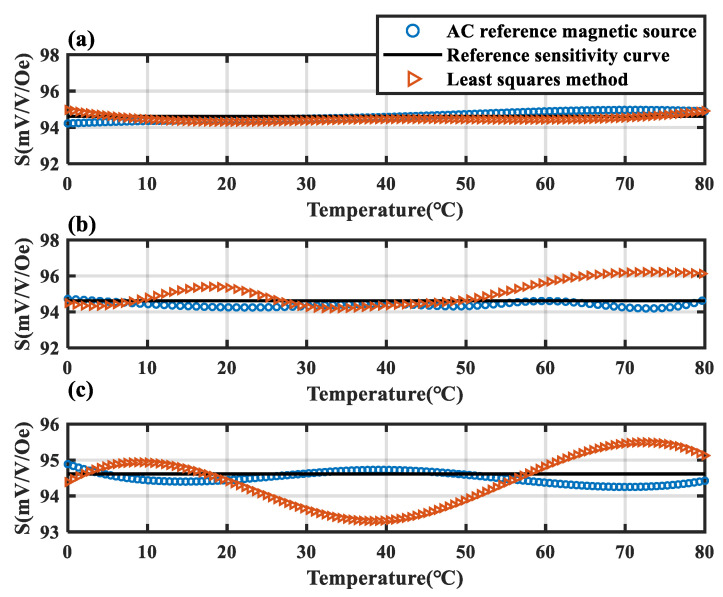
Comparison of the compensation results of different temperature compensation methods. (**a**) Compensation results of the sensitivity curves for the first experiment between the conventional method and the proposed method. (**b**) Compensation results of the sensitivity curves of the second experiment for the conventional method and the proposed method. (**c**) Compensation results of the sensitivity curves for the third experiment between the conventional method and the proposed method.

The black line is the sensitivity reference line measured at 0 °C. It can be observed that the linearity and repeatability of the TMR sensitivity curves measured in the three experiments are relatively poor. Following compensation with the AC reference magnetic source, the sensitivity of the TMR sensor remains stably at the reference sensitivity measured at 0 °C under variable temperature conditions. The sensitivity error of the compensated TMR magnetic sensor is constrained within a range of −0.41% to 0.28%. Using the data from the green curve in Figure 9 as the sample, the least squares method in traditional software compensation was applied to fit the temperature compensation curve. The fitted curve was then used to compensate the remaining two curves. The compensation results were compared with the compensation effect of the temperature compensation method under the reference magnetic source, as shown in Figure 10.

The yellow and blue curves represent the compensation results for TMR sensitivity using the least squares method and the reference magnetic source-based method, respectively. In Figure 10a, the yellow curve shows the compensation of the sample’s own curve based on the fitted least squares curve, showing a compensation performance comparable to the proposed method. However, Figure 10b,c reveal that the least squares method performs poorly in compensating the other two curves, indicating that it is ineffective for achieving high-precision compensation on sensitivity curves with poor repeatability. In contrast, the proposed method achieves excellent compensation across all test curves. The root mean square error (RMSE) was used to evaluate the repeatability of the sensitivity curves after temperature compensation. The RMSE of the two TMR sensitivity curves, following compensation using the least squares method, changed from 0.84 to 0.86, and the RMSE of the two sensitivity curves compensated by the reference magnetic source method decreased from 0.84 to 0.21. It can be observed that after compensation using traditional methods, the repeatability of the sensitivity curve did not show significant improvement. However, with the compensation method based on an AC reference magnetic source, the repeatability of the sensitivity curve was significantly enhanced. This demonstrates that the reference magnetic source-based temperature compensation method significantly outperforms traditional approaches, providing higher repeatability of sensitivity after compensation, which confirms the accuracy of the compensation method.

## 5. Conclusions

In conclusion, we proposed a temperature compensation method based on an AC reference magnetic source, where the real-time sensitivity of a TMR sensor was calculated by the change in the output of the sensor caused by the reference magnetic field, and the temperature drift of the sensitivity was compensated. Finally, a temperature compensation system for TMR magnetic sensors was designed to verify the effectiveness of the method. The results show that the temperature compensation method based on an AC reference magnetic source effectively reduced the TMR sensitivity drift coefficient from 985.39 ppm/°C to 59.08 ppm/°C. After compensation, the sensitivity error was confined to within ±0.41%, and the root mean square error of the compensated sensitivity curves dropped from 0.84 to 0.21. Furthermore, the compensation principle and process are completely independent of specific temperature values. As a result, the compensation module for sensitivity drift does not require dedicated temperature sensors, reducing accuracy errors caused by temperature inconsistencies among sensor components. This method establishes an efficient, real-time temperature compensation mechanism, significantly enhancing the performance of TMR magnetic sensors in high-precision measurement scenarios. However, our study does not include the suppression of zero-point drift caused by temperature. In the future, we will continue to explore the mechanism of zero-point temperature drift and work on temperature compensation for the zero-point drift of TMR magnetic sensors.

## Figures and Tables

**Table 1:** True sensitivity values and compensation sensitivity values of the TMR sensor in the range of 0–120 °C.

Temperature T (°C)	Actual Value S_DC_ (mV/V/Oe)	Compensation Value S_AC_ (mV/V/Oe)	Output S_O_ (mV/V/Oe)
0	94.61	94.22	94.22
20	92.03	91.84	94.43
40	90.24	90.21	94.58
60	88.28	88.53	94.88
80	86.83	87.08	94.89
100	86.08	85.73	94.22
120	84.10	83.85	94.33

## Data Availability

The original contributions presented in this study are included in the article.
